# Medical Miscellany

**Published:** 1853-09

**Authors:** 


MEDICAL MISCELLANY.

One Dr. Wm. Turner lately presented a petition to the Legislature of New York, praying that physicians may be restrained from drawing blood. That body, by some unaccountable negligence, omitted to pass a law to that effect.——The Sandwich Islands are becoming depopulated. In a given time the number of deaths was to that of births as *six* to *one*.——The post mortem examination of a young lady in Paris, disclosed the fact that three of the ribs had encroached upon the liver, to such an extent as to produce death, and she perished of tight lacing.——Drs. Wood and Bache are on a professional visit from this country, to Europe.——The deaths in London average about 1,000 every week.——Mrs. Pusher died at Antigonish, N. S., aged 105 years, leaving 157 descendants.——A Cincinnati druggist, recently, while asleep, swallowed a gold plate npon which false teeth had been inserted. It lodged a short distance below his palate, and will neither go up or down. It caused him great pain, and it was feared that lock-jaw would ensue.——So common has it become to prosecute surgeons for malpractice, that Dr. Josiah Crosby, an eminent surgeon in Manchester, N. H., refused to dress a fractured limb, recently, unless the patient would place himself under bonds not to prosecute, in the event the limb should not be perfect.——Mr. Nourse, of Andover, Mass., weighs 388 lbs.; a Dr. Brown, of N. Y., weighs 408. ——A respectable woman, living in Eynesburg, who had been totally blind for twenty years, fell down stairs, and the shock caused by the fall resulted in the complete recovery of her sight: so says the *Cambridge Press*.——The Yellow Fever, in a modified and aggravated form, rages with fearful violence at New Orleans this season. The number of interments have averaged from 225 to 250 daily.

